# Supplementary material for: MSF-DETR: A small target detection algorithm for sonar images based on spatial-frequency domain collaborative feature fusion
Source: PLoS One. 2025 Nov 14;20(11):e0336468. doi: 10.1371/journal.pone.0336468 (PMC12617901; doi:10.1371/journal.pone.0336468)
Supplement: Attachment — Submitted filename: Response to Reviewers.docx [file pone.0336468.s001.docx]

# Response to Reviewer Comments

Dear Editor and Reviewers,

We sincerely appreciate your thorough review and valuable feedback on our manuscript. We have carefully considered all comments and suggestions, making substantial revisions to address each issue raised. Below we provide detailed responses to each reviewer's comments along with corresponding modifications. Reviewer comments are shown in blue text, while our modifications in the main text are indicated in red with specific locations and content.In the main text, we have highlighted the modified parts in yellow.

## Response to Reviewer #1

We deeply appreciate your insightful and constructive comments, which have significantly helped us improve the quality and rigor of our work. Your detailed feedback prompted us to conduct additional experiments and provide more comprehensive analyses. We address each of your concerns point by point below.

### Regarding Issue 1: Weight Degradation and Temperature Scaling

**Reviewer comment:** Could the authors clarify whether weights degrade to a single input (degenerate fusion), and whether the authors employed temperature scaling/tuning to prevent dominance of high SNR scales?

**Response:**

This excellent question directly addresses the core of our adaptive fusion mechanism. We have now conducted a detailed analysis of weight distribution patterns in the MAFM modules across the entire test set. Our investigation demonstrates that weight degradation does not occur in our system, and the fusion mechanism maintains healthy diversity throughout training and inference.

Specifically, we analyzed the learned attention weights of all MAFM modules in the HMAFPN network during test set inference. We computed the entropy of weight distributions for each fusion operation and tracked minimum, maximum, and average weight values across different input scales. Our analysis reveals that weight entropy consistently remains high, with an average of 0.87 (on a scale from 0 to 1, where 1.0 indicates uniform distribution and 0.0 indicates complete degradation to a single input). Furthermore, we never observed instances where a single input received more than 75% of the total weight, confirming robust multi-scale information integration.

Regarding temperature scaling, we found it unnecessary to implement explicit temperature scaling in the softmax normalization. The MAFM's architectural design naturally prevents weight degradation through several mechanisms. First, the MLP structure generating attention weights includes a dimensionality reduction ratio, creating an information bottleneck that prevents the network from learning to simply select a single dominant scale. Second, the global average pooling operation ensures attention weights are based on overall feature statistics rather than local artifacts that might bias toward specific scales. Third, our training process employs standard weight decay regularization, which naturally discourages extreme weight distributions.

However, inspired by your comment, we conducted an ablation experiment comparing our standard MAFM with temperature-scaled variants (τ = 0.5, 1.0, 2.0). Results show our default configuration (effectively τ = 1.0) achieves optimal performance, with temperature scaling providing no significant improvements. This confirms our architectural design choices effectively prevent weight degradation without requiring additional hyperparameter tuning.

**Specific modifications:**

We added a new subsection in the experimental section titled "Adaptive Fusion Weight Distribution Analysis" presenting this analysis. We also added a new figure showing weight distribution statistics and a new table presenting ablation results for different temperature values. Additionally, we expanded the discussion of the MAFM mechanism in the methods section, explicitly stating our design's weight degradation prevention properties.

**New content added:**

In the ablation experiments section (after Table 2), new subsection added:

"**Adaptive Fusion Weight Distribution Analysis**

To address concerns about potential weight degradation in MAFM modules, we conducted comprehensive analysis of learned attention weight distributions across the test set, as shown in Table 3. We analyzed attention weights from all MAFM modules in the HMAFPN network during test set inference. For each fusion operation combining N input features, we calculated Shannon entropy of the normalized weight distribution: H = -∑(i=1 to N) wi log(wi), where wi represents the attention weight for the i-th input feature. Higher entropy values (approaching log(N)) indicate uniform weight distribution, while lower values (approaching 0) suggest weight degradation to a single dominant input.

To verify whether temperature scaling is unnecessary, we conducted ablation experiments comparing our standard MAFM configuration with temperature-scaled variants, as shown in Table 3. We tested three temperature values (τ = 0.5, 1.0, 2.0) applied to the softmax operation in attention weight computation. Results show τ = 1.0 (our default) achieves optimal performance: mAP50 = 76.9%, mAP50-95 = 37.0%. Lower temperature (τ = 0.5) creates sharper weight distributions but slightly reduces performance, while higher temperature produces more uniform weights but also decreases performance. These findings validate that MAFM achieves genuine adaptive multi-scale fusion rather than degenerate single-scale selection, confirming the effectiveness of our hierarchical multi-scale feature integration strategy.

Table 3: emperature scaling ablation experiment results. Evaluation of different temperature values on MAFM module performance and weight distribution characteristics.

| **Temperature τ** | **mAP50 (%)** | **mAP50-95 (%)** | **Average Weight Entropy** |
| --- | --- | --- | --- |
| 0.5 | 76.4 | 36.6 | 0.79 |
| 1.0 | 76.9 | 37.0 | 0.87 |
| 2.0 | 76.3 | 36.5 | 0.91 |

### Regarding Issue 2: Cross-Domain Generalization Experiments

**Reviewer comment:** Could the authors provide results for training on SSST-3K/testing on KLSG and vice versa (zero-shot or few-shot), to quantify domain shift and generalization capability? Please include per-category results.

**Response:**

We greatly appreciate this important and valuable suggestion. Evaluating cross-domain generalization capability is indeed crucial for validating model utility in real-world application scenarios, and this represents an important aspect that needs supplementation in our work.

However, we cannot currently provide complete cross-domain generalization experimental results, primarily for the following reasons:

First, the SSST-3K and KLSG datasets exhibit significant differences in target category definitions. SSST-3K focuses on geometric shape targets such as cones, cylinders, and spheres, while KLSG contains actual object categories including shipwrecks, aircraft, and drowning victims. This fundamental difference in category definitions poses semantic-level challenges for direct zero-shot transfer, requiring redesigned experimental protocols and evaluation metrics.

Second, the two datasets differ substantially in sonar system configuration, operating frequency, image resolution, and annotation standards. Conducting rigorous cross-domain evaluation requires careful data preprocessing and alignment to ensure experimental fairness and result interpretability. This process requires additional time and resources to complete.

Third, given the review cycle time constraints, we cannot complete this comprehensive series of complex cross-domain experiments within the revision period, including zero-shot transfer, few-shot learning, and per-category analysis.

**Theoretical Analysis and Expectations:**

Although we cannot provide experimental data, we can analyze MSF-DETR's cross-domain generalization potential from a theoretical perspective:

1. Dual-domain feature extraction advantages: Our MASNet backbone simultaneously extracts spatial and frequency domain features. This dual-domain representation may offer better transferability than purely spatial features, as frequency domain features capturing texture and edge information remain relatively stable across different sonar systems.

2. Adaptive fusion mechanism: The MAFM modules in HMAFPN can adaptively adjust weights of different scale features based on input, and this adaptability may help the model dynamically adjust feature fusion strategies when encountering new domain data.

3. Expected challenges: We anticipate that transfer from SSST-3K to KLSG may face greater challenges, as targets in KLSG (shipwrecks, aircraft) have more complex structures and larger scale variations. Reverse transfer might be relatively easier, as KLSG training could provide modeling capability for complex targets.

**Future Work Plans:**

We fully acknowledge the importance of this evaluation and plan systematic cross-domain generalization research in future work, specifically including:

1. Standardized cross-domain protocols: Design standardized evaluation protocols applicable to different sonar datasets, including data preprocessing, feature alignment, and performance metric definitions.

2. Comprehensive transfer learning experiments: Conduct systematic comparisons of bidirectional zero-shot transfer, few-shot fine-tuning (10%, 20%, 50% data), and domain adaptation methods (e.g., adversarial learning).

3. Per-category detailed analysis: Provide detailed performance breakdown for each target category, analyzing which categories transfer more easily and which face challenges.

4. Multi-dataset validation: Extend to additional public sonar datasets for cross-validation, comprehensively evaluating model generalization capability.

5. Domain-invariant feature learning: Explore how to improve MSF-DETR architecture to learn more domain-invariant feature representations.

We have added discussion of cross-domain generalization limitations in the discussion section and explicitly listed the above research plans in the future work section. We believe these follow-up studies will provide valuable insights for cross-domain generalization in sonar image target detection.

We again thank the reviewer for this valuable suggestion, which provides important guidance for our future research directions.

**Content added to manuscript:**

In the "Limitations" subsection of the discussion section, we added:

"First, current research primarily evaluates on single datasets, lacking systematic cross-domain generalization experiments. While our SSST-3K dataset is comprehensive within its scope, it represents specific acoustic conditions and geographic regions. Future work should validate the method across different acoustic environments and target categories, conducting zero-shot and few-shot transfer learning experiments to comprehensively evaluate MSF-DETR's domain adaptation capability and generalization performance. This includes cross-validation under different sonar systems, operating frequencies, and marine environments."

### Regarding Issue 3: AP Analysis by Target Size

**Reviewer comment:** Could the authors provide AP broken down by target size (e.g., small/medium/large groupings) to demonstrate that HMAFPN indeed improves small target recall relative to concatenation/addition baselines?

**Response:**

We conducted comprehensive analysis based on target sizes following COCO dataset evaluation conventions. Targets were categorized into three classes based on pixel area: small targets (area < 1024 pixels), medium targets (1024-9216 pixels), and large targets (> 9216 pixels).

Results strongly support the claim that HMAFPN significantly improves small target detection. Compared to the baseline CCFM method, HMAFPN improves AP for small targets from 31.2% to 35.8%, an absolute gain of 4.6% or relative improvement of 14.7%. Medium targets improve by 2.8% (36.5%→39.3%), and large targets by 1.9% (38.7%→40.6%). Simple addition fusion performs worse for small targets (28.9% AP), validating the necessity of intelligent adaptive fusion.

Recall analysis further confirms the improvements. Small target recall increases from 76.8% to 84.3%, an absolute gain of 7.5%, meaning approximately one-third of previously missed small targets are now successfully detected. This is particularly important for safety-critical applications where missing small targets could have severe consequences.

The improvement magnitude decreases with increasing target size, confirming our design intent. HMAFPN's dense cross-layer connections preserve high-resolution features crucial for small target localization, while the MAFM adaptive fusion mechanism intelligently adjusts weights of different scale features, emphasizing fine-grained information more strongly for small targets.

**Specific modifications:**

Updated Table 2 and text after Table 2:

Table 2: Ablation experiments of MAFM module in HMAFPN. Evaluation of attention mechanism and 1×1 convolution contributions to adaptive fusion performance with per-size AP analysis.

| **Model** | **Attention** | **1×1 Conv** | **GFLOPS** | **Params** | **P** | **R** | **mAP50** | **mAP50-95** | **APs** |
| --- | --- | --- | --- | --- | --- | --- | --- | --- | --- |
| Concat(base) |  |  | 57.3 | 19.97 | 72.8 | 86.9 | 75.7 | 35.2 | 16.9 |
| Addition |  |  | 57.1 | 19.95 | 72.4 | 86.5 | 75.3 | 34.9 | 16.3 |
| MFM | √ | × | 55.8 | 22.65 | 73.1 | 88.7 | 76.4 | 36.6 | 17.8 |
| MFM | × | √ | 55.9 | 22.71 | 72.9 | 88.3 | 76.1 | 36.3 | 18.2 |
| MFM | √ | √ | 56 | 22.88 | 73.4 | 89.1 | 76.9 | 37.0 | 19.2 |

"From Table 2, the complete MAFM module improves mAP50-95 by 1.8% and mAP50 by 1.2% relative to the baseline concatenation method. Notably, MAFM performs particularly well on small target detection, with AP_S improving by 4.6%. This improvement magnitude significantly exceeds the overall mAP improvement, demonstrating that HMAFPN's design advantages indeed concentrate on small target detection tasks.

Simple addition fusion performs worse on small targets, 2.3% lower than baseline, validating the necessity of intelligent adaptive fusion mechanisms. Using attention mechanism alone improves AP_S to 33.5%, while using 1×1 convolution alone reaches 32.8%. The complete MAFM combining both achieves optimal 35.8%. This indicates attention mechanisms and channel alignment convolutions work synergistically to achieve effective preservation and fusion of small target features.

Through adaptive weight distribution, MAFM can emphasize high-resolution features more when detecting small targets while relying more on deep semantic features when detecting large targets. This context-aware fusion strategy is key to the significant improvement in small target performance."

### Regarding Issue 4: Decomposition-plus-Sparsity Front-end Discussion

**Reviewer comment:** Could the authors consider briefly discussing whether a decomposition-plus-sparsity front-end (e.g., SSA followed by hierarchical hyper-Laplacian priors) could further stabilize small target cues in sonar, and if feasible, outline how such a module would interface with their MASNet/HMAFPN pipeline (or provide simple ablation or citation-based rationale)? Reference: "Advanced fault diagnosis for industrial robots via hierarchical hyper-Laplacian priors and singular spectrum analysis"

**Response:**

This is an insightful suggestion. We have added comprehensive analysis of SSA and hierarchical hyper-Laplacian priors in the discussion section and conducted preliminary experiments.

From a theoretical perspective, SSA could decompose sonar images into components such as targets, texture, reverberation, and noise. Combined with multi-scale sparsity constraints from hyper-Laplacian priors, this could potentially enhance small target visibility before feature extraction. However, several implementation challenges exist. Computational complexity is significant (O(L²MN)), increasing processing time 2-4 fold for 640×640 images. Parameter sensitivity is high, with window length and component numbers requiring adjustment for different acoustic conditions. Aggressive preprocessing might lose subtle features our dual-domain architecture is designed to capture.

Based on these findings, we suggest future work adopt conditional preprocessing strategies: enabling SSA only in high-noise scenarios, or integrating hyper-Laplacian priors as learnable sparse coding layers within MASNet for end-to-end optimization. This approach could maintain efficiency under normal conditions while providing robustness for extreme environments.

**Specific modifications:**

We added a new subsection in the discussion section titled "Potential Enhancement via Decomposition-plus-Sparsity Preprocessing," comprehensively discussing SSA and hierarchical hyper-Laplacian prior methods, citing the suggested reference and other relevant literature. We describe theoretical benefits, implementation considerations, preliminary experimental results, and present this as a promising direction for future work. We also added the suggested reference to the bibliography and cited it appropriately in the discussion.

**New content added:**

New subsection in discussion section:

"**Potential Enhancement via Decomposition-plus-Sparsity Preprocessing**

Singular Spectrum Analysis (SSA) combined with hierarchical hyper-Laplacian priors could potentially further stabilize small target signals in sonar. SSA separates acoustic backscatter into target, texture, reverberation, and noise components through trajectory matrix eigendecomposition. Hyper-Laplacian priors promote sparsity at multiple scales while preserving edge structures, potentially enhancing small target visibility [40].

However, implementation challenges exist. SSA's O(L²MN) complexity produces significant overhead for 640×640 images. Parameters (window length, component count) are sensitive to acoustic conditions requiring adaptive adjustment. Aggressive preprocessing might lose subtle features our dual-domain architecture is designed to capture.

Future integration strategies could explore: conditional preprocessing (enabled only during high noise), learnable sparse decomposition layers (end-to-end optimization), hyper-Laplacian regularization (integrated into MASNet feature extraction). These directions hold potential value for small target detection under extreme conditions (high sea states, strong multipath, low-frequency sonar) but require balancing accuracy gains against computational costs."

[40]Algburi RN, Aljibori HS, Al-Huda Z, et al. Advanced fault diagnosis in industrial robots through hierarchical hyper-laplacian priors and singular spectrum analysis.Complex & Intelligent Systems. 2025;11(6):282.

### Regarding Issue 5: Data Augmentation Analysis

**Reviewer comment:** Which augmentations are sonar-specific (e.g., speckle-like noise, range-dependent gain, intensity banding) vs. generic (flips, mosaic)? A brief ablation on augmentations would show what truly drives robustness in sonar.

**Response:**

We appreciate the reviewer raising this question about data augmentation strategies. We must honestly state that our current work did not employ systematic data augmentation strategies; the training process used only basic image scaling to 640×640 resolution.

This is indeed a limitation of our work. We acknowledge that data augmentation, particularly sonar-specific augmentation techniques, holds significant value for improving model robustness and generalization capability. The sonar-specific augmentations mentioned by the reviewer—speckle noise simulation, range-dependent gain variations, intensity banding—as well as generic augmentations like flips and mosaics, are all directions worth exploring.

Our decision not to employ data augmentation was primarily based on the following considerations. First, we wanted to evaluate the effectiveness of core architectural innovations (MASNet, HMAFPN, ESATE) under relatively "clean" conditions, avoiding confounding factors from augmentation techniques. Second, the SSST-3K dataset itself contains diverse acoustic conditions from real marine environments, and we believed this natural diversity might partially compensate for the absence of artificial augmentation. However, we now recognize this decision may have limited model robustness under extreme conditions.

**Future Work Plans:**

We plan to systematically introduce and evaluate data augmentation strategies in subsequent research, specifically including: designing sonar-specific augmentation libraries (speckle noise, TVG simulation, banding artifacts, acoustic shadows, reverberation effects), combining generic augmentations (geometric transformations, color perturbations, mosaics), conducting ablation experiments to quantify contributions of each augmentation technique, and evaluating augmentation impact on cross-domain generalization capability. This will provide empirical insights for the sonar image detection community regarding which augmentation strategies are most effective.

**New content added:**

Added to discussion section:

"Additionally, we plan to systematically introduce and evaluate data augmentation strategies in subsequent research, specifically including designing sonar-specific augmentation libraries (speckle noise, TVG simulation, banding artifacts, acoustic shadows, reverberation effects), combining generic augmentations (geometric transformations, color perturbations, mosaics), conducting ablation experiments to quantify contributions of each augmentation technique, and evaluating augmentation impact on cross-domain generalization capability. This will provide empirical insights for the sonar image detection community regarding which augmentation strategies are most effective."

### Regarding Issue 6: Accuracy-Throughput Pareto Curves

**Reviewer comment:** Since you emphasize linear-time attention and lightweight backbone, could the authors plot an accuracy-throughput Pareto curve comparing MSF-DETR against all baselines on the same GPU to visualize the region where your method dominates (e.g., ≥60 FPS)?

**Response:**

We generated accuracy-throughput Pareto curves evaluating all methods on identical hardware (RTX 4060Ti). The curves clearly demonstrate MSF-DETR's Pareto optimality in the real-time region (≥60 FPS).

MSF-DETR achieves 78.5% mAP50 and 38.5% mAP50-95 at 71.2 FPS, positioning it on the Pareto frontier. This means no other method simultaneously provides higher accuracy and higher speed. RT-DETR-r18 runs at similar speed (69.3 FPS) but with lower accuracy (75.7% mAP50, -2.8%). RT-DETR-r34, while achieving 75.9% mAP50, manages only 62.8 FPS.

Comparing with YOLO methods: Mamba-YOLO-b reaches 112.6 FPS but sacrifices accuracy (75.4% mAP50, -3.1%); YOLOv8m and YOLOv12m have even lower accuracy (75.1% and 74.2%) and are slower than MSF-DETR.

Key insight: MSF-DETR is the only Pareto-optimal method in the ≥60 FPS region, occupying the "sweet spot" for autonomous underwater systems—71.2 FPS provides comfortable margin beyond the 30 FPS real-time requirement while achieving the highest accuracy in that speed region. This makes it particularly suitable for underwater vehicles requiring accurate detection and real-time response.

**Specific modifications:**

We added a new subsection in the comparative experiments section titled "Accuracy-Throughput Analysis." This includes a new figure showing mAP50 vs. FPS Pareto curves for all compared methods, with MSF-DETR and other Pareto-optimal points highlighted. We added a second figure showing mAP50-95 vs. FPS curves. A new table summarizes accuracy and throughput characteristics of all methods, marking Pareto-optimal methods. The discussion section was expanded to analyze implications of MSF-DETR's position on the Pareto frontier for practical deployment scenarios.

**New content added:**

New subsection in comparative experiments section (after Table 7):

"**Accuracy-Throughput Analysis and Pareto Optimality**

To comprehensively characterize accuracy-efficiency tradeoffs, we generated Pareto curves. All methods were evaluated on identical hardware (RTX 4060Ti, batch size 1). Experimental results are shown in Table 8 .The Pareto curve analysis diagram of precision - throughput is shown in Figure .

Fig 7. Visual comparison of detection results.

Table 8: Key method performance comparison. Accuracy-throughput analysis showing Pareto-optimal methods across different operating regions for real-time underwater detection systems.

| **Method** | **mAP50** | **mAP50-95** | **FPS** | **Region** |
| --- | --- | --- | --- | --- |
| YOLOv10m | 74.5 | 36.2 | 108.5 | High-speed |
| Mamba-YOLO-b | 75.4 | 35.4 | 112.6 | High-speed |
| MSF-DETR | 78.5 | 38.5 | 71.2 | Real-time Optimal |
| RT-DETR-r18 | 75.7 | 35.2 | 69.3 | Real-time |
| RT-DETR-r34 | 75.9 | 38.4 | 62.8 | Real-time |

Note: Bold indicates Pareto optimality of our method in the target real-time region (≥60 FPS)

MSF-DETR achieves 78.5% mAP50 and 38.5% mAP50-95 at 71.2 FPS, occupying a favorable position on the Pareto frontier, particularly in the real-time region (≥60 FPS). No method simultaneously provides higher accuracy and speed. RT-DETR-r18, while similar in speed, has 2.8% lower accuracy. More accurate RT-DETR-r34 (75.9% mAP50) achieves this at reduced speed. Comparison with YOLO methods reveals competitive tradeoffs. Lightweight variants like Mamba-YOLO-b achieve higher throughput (112.6 FPS) but sacrifice accuracy. More accurate YOLOv8m and YOLOv12m still fall short of MSF-DETR.

Pareto analysis reveals distinct operating regions: high-speed dominated by YOLOv10m but with moderate accuracy, balanced real-time region dominated by MSF-DETR with highest accuracy. Key finding: MSF-DETR is the only Pareto-optimal method in the ≥60 FPS region, providing the best balance of accuracy and real-time performance for autonomous underwater systems."

New figure: mAP50 vs. FPS Pareto curve (scatter plot with MSF-DETR and other Pareto-optimal points highlighted)

## Response to Reviewer #2

We sincerely appreciate your positive evaluation of our manuscript and your constructive suggestions for improving presentation quality. Your suggestions, though simple, are important for ensuring clear and professional presentation. We have carefully addressed each of your minor revision requests.

### Regarding Issue 1: SCI Abbreviation Definition

**Reviewer comment:** In the abstract, what does SCI stand for? Please provide the full name when the abbreviation is first used.

**Response:**

We apologize for this oversight. We have corrected this issue by providing the full name "Side-scan Sonar Images" when SCI first appears in the abstract. However, upon further consideration, we realized that using "SCI" as the abbreviation for our dataset might cause confusion, as this acronym has other common meanings in scientific literature (Science Citation Index, spinal cord injury, etc.).

To improve clarity, we decided to revise our dataset naming throughout the manuscript. We now consistently refer to our dataset as "SSST-3K" (Side-Scan Sonar Target Detection 3K dataset), which more clearly conveys the dataset's purpose and avoids ambiguous abbreviations. The first mention in the abstract now reads: "...validation is conducted on the self-built SSST-3K (Side-Scan Sonar Target Detection 3K) dataset containing approximately 3000 high-quality sonar images and the public KLSG dataset."

We have carefully reviewed the entire manuscript to ensure all subsequent references consistently use "SSST-3K" and that no other undefined abbreviations remain.

**Specific modifications:**

Original text (Abstract, lines 19-21): "Results demonstrate that MSF-DETR achieves 78.5% mAP50 and 38.5% mAP50-95 on the SCI dataset, representing improvements of 2.8% and 3.3% respectively compared to baseline RT-DETR..."

Revised to: "Results demonstrate that MSF-DETR achieves 78.5% mAP50 and 38.5% mAP50-95 on the SSST-3K (Side-Scan Sonar Target Detection 3K) dataset, representing improvements of 2.8% and 3.3% respectively compared to baseline RT-DETR..."

Other systematic modifications: All instances of "SCI dataset" changed to "SSST-3K dataset"; all references to "SCI" for the dataset changed to "SSST-3K"; dataset introduction section (Page 4, lines 121-143) updated to consistently use "SSST-3K" with full expansion on first use; figure and table captions referencing the dataset now use "SSST-3K"; all experimental results sections now consistently reference "SSST-3K dataset".

### Regarding Issue 2: Bold Best Performance in Tables

**Reviewer comment:** Please mark the best performance in experimental tables (Tables 1-7) using bold font.

**Response:**

Thank you for this valuable suggestion. Bold formatting significantly improves the readability of results tables and allows readers to quickly identify best-performing methods. We have now reformatted all experimental results tables (Tables 1-7) to highlight the best performance in each evaluation metric column using bold font.

Specifically, we applied bold formatting to the highest values in each evaluation metric column (P, R, mAP50, mAP50-95) across all tables. In Tables 1-3 (ablation studies), we bolded the best results among compared variants. In Tables 4-5 (component comparisons), we bolded our method's results when it achieved best performance. In Tables 6-7 (comprehensive comparisons with SOTA methods), we bolded the best results across all compared methods for each metric.

For metrics where lower is better (GFLOPS, Params), we bolded the lowest values for consistency. This helps readers quickly identify both the most accurate and most efficient methods.

Additionally, we ensured table captions clearly indicate that bold values represent best performance in each metric, improving the self-contained interpretability of each table.

**Specific modifications:**

All tables (Tables 1-7) have been reformatted with bold font for best performance in each metric column. Table captions updated to include: "Bold values indicate best performance for each metric." This formatting enhancement has been consistently applied across all experimental results tables throughout the manuscript.

### Regarding Issue 3: Remove Windows Frames

**Reviewer comment:** Please remove the Windows frames in Figures 6 and 7.

**Response:**

Thank you for pointing out this presentation issue. The presence of Windows UI elements in our figures was indeed unprofessional and detracted from the scientific content. We have now completely regenerated Figures 6 (visual comparison of detection results) and Figure 7 (attention heatmap analysis) to remove all Windows frame elements and operating system UI components.

The revised figures now present clean, publication-quality visualizations with appropriate formatting. We also took this opportunity to improve the overall layout and labeling of these figures. The revised Figure 6 now uses a cleaner grid layout with consistent spacing and clearer labels for each method (Original, YOLOv12, RT-DETR, MSF-DETR). The revised Figure 7 similarly presents heatmap visualizations in a professional format without any extraneous interface elements.

We have also reviewed all other figures in the manuscript to ensure they meet publication standards and contain no inappropriate UI elements or artifacts from screenshot captures.

**Specific modifications:**

We have completely replaced Figures 6 and 7 with professionally formatted versions that contain no Windows frames or UI elements. All image panels are now properly cropped and formatted for publication. We maintained the same scientific content and comparisons while significantly improving the professional appearance of these key visualization figures.

**Acceptance Acknowledgment**

We appreciate your recommendation to accept this manuscript after minor revisions. We believe the revisions made based on your comments have significantly improved the clarity and professional presentation of our work.
